# 2,3,5,6-Tetramethylpyrazine Targets Epithelial-Mesenchymal Transition by Abrogating Manganese Superoxide Dismutase Expression and TGFβ-Driven Signaling Cascades in Colon Cancer Cells

**DOI:** 10.3390/biom12070891

**Published:** 2022-06-25

**Authors:** Young Yun Jung, Chakrabhavi Dhananjaya Mohan, Huiyan Eng, Acharan S. Narula, Ojas A. Namjoshi, Bruce E. Blough, Kanchugarakoppal S. Rangappa, Gautam Sethi, Alan Prem Kumar, Kwang Seok Ahn

**Affiliations:** 1 Department of Science in Korean Medicine, Kyung Hee University, 24 Kyungheedae-ro, Dongdaemun-gu, Seoul 02447, Korea; ve449@naver.com; 2 Department of Studies in Molecular Biology, University of Mysore, Manasagangotri, Mysore 570006, India; mohan@biochemistry.uni-mysore.ac.in; 3 Department of Pharmacology, Yong Loo Lin School of Medicine, National University of Singapore, Singapore 117600, Singapore; huiyaneng@gmail.com; 4 NUS Centre for Cancer Research (N2CR), Yong Loo Lin School of Medicine, National University of Singapore, Singapore 117599, Singapore; 5 Narula Research, Chapel Hill, NC 27516, USA; anarula1@nc.rr.com; 6 Engine Biosciences, 733 Industrial Rd., San Carlos, CA 94070, USA; ojas.namjoshi@enginebio.com; 7 Center for Drug Discovery, RTI International, Research Triangle Park, Durham, NC 27616, USA; beb@rti.org; 8 Institution of Excellence, Vijnana Bhavan, University of Mysore, Manasagangotri, Mysore 570006, India; rangappaks@ioe.uni-mysore.ac.in

**Keywords:** Tetramethylpyrazine, epithelial-mesenchymal transition, MnSOD, TGFβ signaling

## Abstract

Epithelial-mesenchymal transition (EMT) is a crucial process in which the polarized epithelial cells acquire the properties of mesenchymal cells and gain invasive properties. We have previously demonstrated that manganese superoxide dismutase (MnSOD) can regulate the EMT phenotype by modulating the intracellular reactive oxygen species. In this report, we have demonstrated the EMT-suppressive effects of 2,3,5,6-Tetramethylpyrazine (TMP, an alkaloid isolated from Chuanxiong) in colon cancer cells. TMP suppressed the expression of MnSOD, fibronectin, vimentin, MMP-9, and N-cadherin with a parallel elevation of occludin and E-cadherin in unstimulated and TGFβ-stimulated cells. Functionally, TMP treatment reduced the proliferation, migration, and invasion of colon cancer cells. TMP treatment also modulated constitutive activated as well as TGFβ-stimulated PI3K/Akt/mTOR, Wnt/GSK3/β-catenin, and MAPK signaling pathways. TMP also inhibited the EMT program in the colon cancer cells-transfected with pcDNA3-MnSOD through modulation of MnSOD, EMT-related proteins, and oncogenic pathways. Overall, these data indicated that TMP may inhibit the EMT program through MnSOD-mediated abrogation of multiple signaling events in colon cancer cells.

## 1. Introduction

Colon and rectal cancers generally originate in the inner lining of the colon or rectum and protrude as small extra growths termed polyps, which are usually benign. Polyps may become cancerous with the advancement of time. Colon and rectal cancers are often grouped due to the sharing of multiple clinical features. Colorectal cancers have been commonly appearing among the top five lethal cancers and are considered one of the leading types of malignancies with a worldwide mortality rate of 862,000 as per 2018 statistics [1]. Liver metastasis is commonly observed (over 50% cases) in colorectal cancer patients, which makes available treatment regimens ineffective.

Epithelial-mesenchymal transition (EMT) is a complex process that involves the transition of the polarized epithelial cells to attain a mesenchymal phenotype that exhibits increased migratory potential, invasive capability, and apoptotic resistance. In general, epithelial cells interact with the basement membrane to be immobile and alteration in the expression of cell adhesion components, transcription factors, extracellular matrix-degrading enzymes, and cytoskeletal elements results in their transition to mesenchymal phenotype [2]. Numerous studies have provided strong evidence that EMT is crucial for cancer advancement and metastasis in a broad range of human malignancies including colorectal cancer. During EMT, cells dislodge from the primary tumor and attain an invasive nature through the dissolution of the tight and adhering junctions, distortion of apical-basal polarity, and reorganization of the cytoskeletal architecture [3]. In addition, in recent studies, EMT has been studied in the form of hybrid EMT, which appears when cancer cells adapt to the more stressful environment for proceeding to metastasis [4]. Moreover, EMT can be triggered by various signaling proteins and growth factors including transforming growth factor β (TGFβ) [5], epidermal growth factor (EGF) [6], cytokines, and many more [7]. TGFβ pathway can serve as an oncogenic as well as tumor-suppressor depending on the tumor grade. It presents tumor-suppressive functions in the early stages of cancer and becomes oncogenic with the advancement in tumor progression due to its ability to promote cell division and EMT in several types of human cancers [8,9]. Moreover, TGFβ also engages in the promotion of metastatic signaling events such as PI3K/Akt/mTOR, Wnt/GSK3/β-catenin, and MAPK pathways [10,11,12,13]. Therefore, inhibition of TGFβ-stimulated EMT could be a potential therapeutic strategy to counteract the cancer progression.

Manganese superoxide dismutase (MnSOD) is a manganese-dependent-mitochondrial enzyme that detoxifies reactive oxygen species (particularly converts superoxide (O^2−^) to hydrogen peroxide (H_2_O_2_)) to maintain the homeostatic condition of the cell and it is rightly regarded as the “Guardian of the Powerhouse” [14]. We have previously demonstrated that MnSOD serves as a switch between EMT and mesenchymal-epithelial transition (MET)-associated phenotype by regulating the redox environment of the cell [15]. Notably, overexpression of miR-212 (a miRNA that directly targets MnSOD mRNA) reduced the levels of MnSOD, which resulted in the blockade of EMT in colorectal cancer cells [16]. It was also demonstrated that MnSOD is essential for the reduction of epithelial markers and elevation of mesenchymal markers in colorectal cancer cells. Additionally, an inverse correlation was observed between the levels of miR-212 and MnSOD protein in colorectal tumor samples [16]. Qiu and colleagues reported that the blockade of MnSOD/FoxM1 signaling in head and neck squamous cell carcinoma cells results in the inhibition of expression of EMT-related transcription factors and MMP-2 [17]. These studies emphasize the importance of MnSOD in the promotion of EMT in human cancers.

2,3,5,6-Tetramethylpyrazine (TMP) is a bioactive alkaloid (also known as ligustrazine) isolated from Chuanxiong (*Ligusticum Wallichii*), which has been traditionally used for the treatment of cardiovascular diseases in Chinese medicine. Some studies have demonstrated the anticancer effects of TMP in various cancer models. Zhou and colleagues demonstrated that TMP imparts cytotoxicity in prostate cancer cells through the inactivation of the DPP10-AS1/CBP/FOXM1 signaling pathway [18]. In another study, TMP was found to suppress cell proliferation and cell migration through downregulation of FOXM1 in prostate cancer cells [19]. Yu and colleagues reported that TMP reduces the expression of CXCR4 to suppress the growth of C6 gliomas [20]. TMP triggered the generation of reactive oxygen species (ROS) to induce anticancer effects in hepatocellular carcinoma [21]. The depletion of ROS suppressed the TMP-induced growth-inhibitory effects in hepatocellular carcinoma cells [21]. Additionally, TMP significantly reversed multidrug resistance in bladder cancer cells by altering the expression of MRP1, GST, Bcl-2, and Topo-II [22]. Although several studies report the anticancer property of TMP, a precise molecular mechanism is yet to be demonstrated. Herein, we have attempted to explore the effect of TMP on the expression of MnSOD and the EMT process in colon cancer cells.

## 2. Materials and Methods

### 2.1. Reagents

Tetramethylpyrazine hydrochloride (TMP) was obtained from Narula Research, LLC (Chapel Hill, NC, USA). The stock solution (100 mM) of TMP was prepared in water and kept at −20 °C. The stock is diluted in cultured media for in vitro experiments as and when required. Tris-base, glycine, sodium chloride, SDS, and bovine serum albumin (BSA) were purchased from Sigma-Aldrich (St. Louis, MO, USA). Alexa Fluor^®^ 594 donkey anti-mouse IgG (H + L) and Alexa Fluor^®^ 488 donkey anti-goat IgG (H + L) were procured from Life Technologies (Grand Island, NY, USA). Antibodies against MnSOD, fibronectin, vimentin, MMP-9, N-cadherin, α-tubulin, β-actin, and Lamin B were procured from Santa Cruz Biotechnology (Dallas, Texas, USA). Antibodies against p-PI3K (Tyr458), PI3K, p-Akt (Ser473), Akt, p-mTOR (Ser2448), mTOR, β-catenin, Wnt3a, p-GSK3β (Ser9), GSKβ, and FZD-1 antibodies were procured from Cell Signaling Technology (Danvers, MA, USA). An anti-p-GSK-3β antibody was procured from Abcam (Cambridge, UK). Forty-eight-well Chamber 3.2 mm diameter wells and 8 μm pores polycarbonate filters were purchased from Neuro Probe Inc (Gaithersburg, MD, USA).

### 2.2. Cell Lines and Culture Conditions

Human colon adenocarcinoma (HCT-116, SNU-C2A, and HT-29) cells were obtained from Korean Cell Line Bank (Seoul, Korea). These cells were propagated in RPMI-1640 medium supplemented with 10% FBS and 1% antibiotic solution.

### 2.3. MTT Assay

The viability of the untreated and TMP-treated cells was measured using the MTT assay as reported earlier [23,24,25]. Cells (1 × 10^4^ cells/well) were seeded on 96-well plates and treated with TMP (0, 2.5, 5, 10 μM) for 24 h. After 24 h of treatment, 30 μL of MTT solution (2 mg/mL) was added, followed by incubation for 2 h and the addition of lysis buffer. Cell viability was examined using VARIOSKAN LUX (Thermo Fisher Scientific Inc, Waltham, MA, USA) at 570 nm.

### 2.4. Western Blot Analysis

The protein expression levels were evaluated by Western blot analysis using protein-specific antibodies as described previously [26,27]. Cells (5 × 10^5^ cells/well) were seeded and treated with the various indicated concentrations of TMP and TGFβ. The cells were harvested, and whole-cell lysates were prepared. An equal amount of proteins was resolved using a sodium dodecyl sulfate-polyacrylamide gel electrophoresis (SDS-PAGE) and transferred to a nitrocellulose membrane. The membrane was then blocked with 5% skimmed milk in 1 × TBST (1 × TBS with 0.1% Tween 20). The membrane was probed with specific antibodies at 4 °C for at least 8 h and washed using 1 × TBST before incubation with secondary antibodies at room temperature for 1 h. The specific bands from the membranes were detected by enhanced chemiluminescence (ECL) kit (EZ-Western Lumi Femto, DOGEN)

### 2.5. RT (Reverse Transcription)-PCR

Total RNA was extracted from the untreated and TMP-treated cells and desired RNA is reverse-transcribed, and transcripts are analyzed as indicated in our previous reports [28,29]. An equal amount of total RNA was reverse-transcribed into cDNA, then RT-PCR was performed to evaluate the expression of fibronectin, vimentin, N-cadherin, and E-cadherin using superscript reverse transcriptase and Taq polymerase (TAKARA, Tokyo, Japan). The pairs of forward and reverse primer sets were used as follows: Fibronectin, 5′-CTCTGAATCCTGGCATTGGT-3′, and ATGATGAGGTGCACGTGTGT-3′. Vimentin, 5′-AGATGGCCCTTGACATTGAG-3′, and 5′-TGGAAGAGGCAGAGAAATCC-3′. N-cadherin, 5′-ATTGTGGGTGCGGGGCTTGG-3′ and 5′-GGGTGTGGGGCTGCAGATCG-3′. E-cadherin, 5′-TGCCCAGAAAATGAAAAAGG-3 and 5′-GTGTATGTGGCAATGCGTTC-3′. Fibronectin was amplified at 94 °C for 5 min, 94 °C for 30 s, 55 °C for 30 s, 72 °C for 30 s with 35 cycles, and extension at 72 °C for 7 min. Vimentin was amplified at 94 °C for 5 min, 94 °C for 30 s, 58 °C for 30 s, 72 °C for 30 s with 35 cycles, and extension at 72 °C for 7 min. N-cadherin was amplified at 94 °C for 5 min, 94 °C for 30 s, 60 °C for 30 s, 72 °C for 30 s with 33 cycles, and extension at 72 °C for 7 min. E-cadherin was amplified at 94 °C for 2 min, 94 °C for 15 s, 58 °C for 30 s, 72 °C for 1 min with 30 cycles, and extension at 72 °C for 5 min. Glyceraldehyde-3-phosphate dehydrogenase (GAPDH) was used as a control.

### 2.6. Real Time-PCR for Occludin and E-cadherin

An equal amount of total RNA was reverse-transcribed into cDNA, then Real Time-PCR was performed to evaluate the expression of occludin and E-cadherin. Using a StepOne real-time PCR instrument (Applied Biosystems, Foster City, CA, USA) with SYBR Green kit (Applied Biosystems^®^, Waltham, MA, USA), real-time-PCR reaction mixtures were prepared (final volume, 10 μL). The pairs of forward and reverse primer sets were used as follows: occludin, 5′-TCAGGGAATATCCACCTATCACTTCAG-3′, and 5′-CATCAGCAGCAGCCATGTACTCTTCAC-3′. E-cadherin, 5′-TGCCCAGAAAATGAAAAAGG-3′ and 5′-GTGTATGTGGCAATGCGTTC-3′. Glyceraldehyde-3-phosphate dehydrogenase (GAPDH) was used as a control. After the PCR reaction, we analyzed the values of cycle threshold (Ct) with fold change (number of fold difference) of different microbial populations relative to control without additives. Relative quantification = 2−ΔCt (Target) −ΔCt (Control), where Ct represents the threshold cycle.

### 2.7. Immunocytochemistry

HCT-116, SNU-C2A, and HT-29 cells were treated with TMP for 24 h and analyzed for distribution of the protein-of-interest using immunocytochemistry analysis as elaborated in our previous reports [30,31]. Untreated and TMP-treated cells were subjected to washing with 1 × PBS and fixation with paraformaldehyde. The fixed cells were treated with 0.2% triton-X-100 for 10 min followed by blocking with BSA (5%) solution. These cells were incubated with primary antibodies overnight at 4 °C against protein-of-interest (MnSOD, fibronectin, and E-cadherin). Subsequently, cells were washed and incubated for 1 h at room temperature with fluorescently labeled antibodies, and cells were again washed and stained with DAPI for 3 min. At last, cells were analyzed by Olympus FluoView FV1000 confocal microscope (Tokyo, Japan).

### 2.8. Real-Time Cell Proliferation Analysis

To quantify cell growth, background hindrance was quantified in culture medium per well as described earlier [32]. Later HCT-116, SNU-C2A, and HT-29 cells were seeded on 16-well E-plates and incubated in Roche xCELLigence Real-Time Cell Analyzer (RTCA) DP instrument (Roche Diagnostics GmbH, Germany). TMP (10 μM)- and TGFβ (10 ng/mL)-treated cell index values were measured every 15 min time intervals.

### 2.9. Cell Invasion Assay

To find out the effect of TMP on cell invasion, we performed an invasion assay using the Boyden chamber as reported previously [33].

### 2.10. Gelatin Zymography

Gelatinolytic activity of MMP-2 and MMP-9 was evaluated by gelatin zymography. HCT-116, SNU-C2A, and HT-29 (1 × 10^4^ cells/well) cells were treated with TMP (10 μM) for 24 h. The supernatants were concentrated and prepared in equal amounts for gelatin zymography. Samples were separated on 0.1% gelatin containing 10% SDS-PAGE gel. Gels were washed with 2.5% Triton X-100 for 1 h and incubated in zymo-reaction buffer at 37 °C, 5% CO_2_ conditions overnight. Next, gels were stained with coomassie brilliant blue (7% glacial acetic acid, 40% methanol, 0.25% Coomassie Brilliant Blue R250) and then destained with destaining buffer (10% glacial acetic acid, 10% methanol) until the band was observed.

### 2.11. Wound Healing Assay

HCT-116, SNU-C2A, and HT-29 (6 × 10^5^ cells/well) cells were seeded on a 6-well plate in a medium without serum, and a wound-healing assay was performed as per the previously published protocol [34,35]. When cell density attained about 80%, wounds were made using the sterile pipette tip. Cells were treated with TMP (10 μM) and TGFβ (10 ng/mL) for 24 h. At 0 h and 24 h, the gap of the wound was measured, and the graph was plotted.

### 2.12. Transfection Experiments with MnSOD Overexpressing Plasmid

HCT-116 (5 × 10^4^ cells/well) cells were seeded on a 12-well plate in a medium without serum and transfected with pcDNA3-MnSOD and pcDNA (300 ng) for 24 h by iN-fect™ in vitro Transfection Reagent (iNtRON Biotechnology, Seongnam, Korea). After transfection, cells were treated with TMP (10 μM) for 24 h or 6 h.

### 2.13. Transfection Experiments with MnSOD siRNA

HCT-116 (5 × 10^4^ cells/well) cells were seeded on a 12-well plate in a medium without serum and transfected with MnSOD siRNA and scrambled-siRNA (50 nM) for 24 h by iN-fect™ in vitro Transfection Reagent (iNtRON Biotechnology, Seongnam, Korea). After transfection, cells were treated with TMP (10 μM) for 24 h.

### 2.14. Statistical Analysis

All the values are represented as the mean ± standard deviation. Statistical significance of the data compared with the untreated control was determined using the Student’s unpaired *t*-test. Significance was set at * *p* < 0.05, ** *p* < 0.01, and *** *p* < 0.001.

## 3. Results

### 3.1. TMP Regulates the Expression of MnSOD and EMT-Related Proteins in Colon Cancer Cells

**Figure 1:**
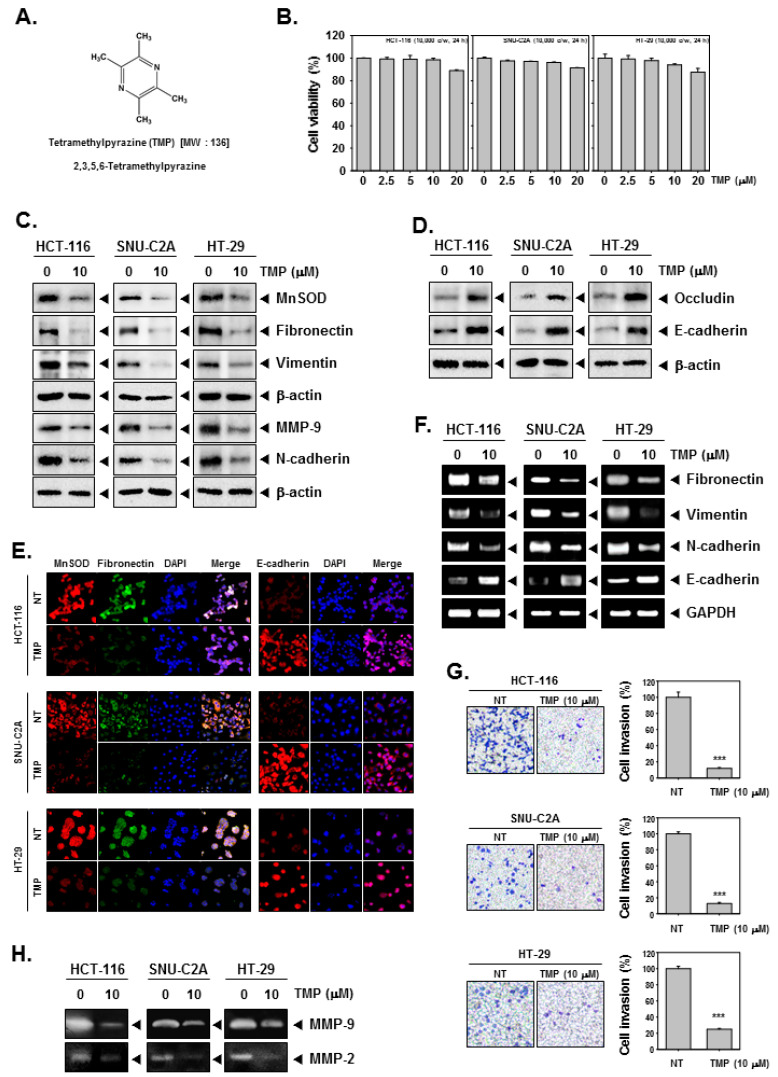
Effects of TMP on EMT in HCT-116, SNU-C2A, and HT-29 cells. (**A**) The chemical structure of TMP. (**B**) HCT-116, SNU-C2A, and HT-29 cells (1 × 10^4^ cells/well) were treated with different doses of TMP for 24 h, and cell viability was quantified. (**C**,**D**) HCT-116, SNU-C2A, and HT-29 cells were exposed to TMP (10 μM) for 24 h. Whole-cell lysates were used to evaluate the expression of indicated proteins by Western blot analysis. (**E**) HCT-116, SNU-C2A, and HT-29 cells were incubated with 10 μM of TMP for 24 h. The expression of indicated proteins was analyzed using immunocytochemistry. (**F**) HCT-116, SNU-C2A, and HT-29 cells were incubated with TMP (10 μM) for 24 h. Total RNA was extracted from cells and transcribed into cDNA to evaluate the expression of indicated transcripts. GAPDH was used as a loading control. (**G**) HCT-116, SNU-C2A, and HT-29 cells were incubated with 10 μM of TMP, and cell invasion was analyzed by Boyden chamber assay. (**H**) The activities of both MMP-2 and 9 were evaluated by zymography. All the experiments were repeated at least thrice. *** *p* < 0.001 vs. non-treated (NT) cells.

The chemical structure of TMP is provided in Figure 1A. HCT-116, SNU-C2A, and HT-29 cells were treated with different doses (0, 2.5, 5, 10, 20 μM) of TMP for 24 h, and cell viability was examined using an MTT assay. TMP displayed marginal cytotoxicity against the tested cells (Figure 1B). We examined the effect on EMT using a concentration at which cell viability was minimally affected. Based on the MTT results, we have used TMP at a concentration of 10 μM, which maintained a survival rate of above 90%.

Next, the expression of MnSOD in TMP-treated colon cancer cells was examined using western blotting. TMP (10 μM) significantly reduced the expression of MnSOD in all three cell lines. Since the expression of MnSOD is associated with the regulation of EMT, the expression of mesenchymal markers (fibronectin, vimentin, and N-cadherin), epithelial markers (occludin and E-cadherin), and MMP-9 was examined in three colon cancer cell lines. TMP (10 μM) significantly downregulated the expression of all the tested mesenchymal markers with an increase in the expression of epithelial markers in all the tested cell lines (Figure 1C,D). Moreover, the expression of MnSOD, fibronectin, and E-cadherin was examined in HCT-116, SNU-C2A, and HT-29 cells using immunofluorescence analysis. The expression of MnSOD and fibronectin was downregulated in TMP-treated cells compared to untreated samples in all three tested cell lines, whereas there was a parallel increase in the expression of E-cadherin in the TMP-treated cells compared to untreated samples (Figure 1E). DAPI was used to stain the nucleus and overlay images were shown.

### 3.2. TMP Modulates the mRNA Expression of Epithelial and Mesenchymal Markers in Colon Cancer Cells

The transcript levels of epithelial and mesenchymal markers were investigated in HCT-116, SNU-C2A, and HT-29 cells upon TMP treatment (10 μM). An evident decline in the levels of the transcript of mesenchymal markers and elevation in the levels of epithelial transcripts was observed on treatment with TMP in all the tested cell lines (Figure 1F). These results indicate that the modulation of expression of EMT-related proteins occurs at the level of transcription.

### 3.3. TMP Reduces Invasion of Colon Cancer Cells

It has been understood from the results of previous assays and reports that the expression of MnSOD contributes to EMT phenotype and reduced expression of MnSOD results in the expression of MET markers. The functional effect of TMP-induced inhibition of MnSOD expression was analyzed using invasion assay as described in the methods section. There was a significant reduction in the number of invaded cells upon TMP treatment (Figure 1G) indicating that alteration in the MnSOD expression upon TMP treatment may have a role in the inhibition of invasion of colon cancer cells.

### 3.4. TMP Suppresses MMP-2/9 Activity in the Supernatant of Colon Cancer Cells

To evaluate the MMP-2 and 9 activities, we obtained the supernatant from TMP-treated HCT-116, SNU-C2A, and HT-29 cells. The supernatants were then concentrated and quantified in equal amounts for running on gelatin containing SDS-PAGE gel (10%) followed by the examination of the expression of MMP-2 and 9. The results showed that TMP can effectively reduce the activities of MMP-2 and 9 in all the tested human colon cancer cells (Figure 1H).

### 3.5. TMP Altered the TGFβ-Induced Expression of EMT-Related-mRNA and -Proteins

**Figure 2:**
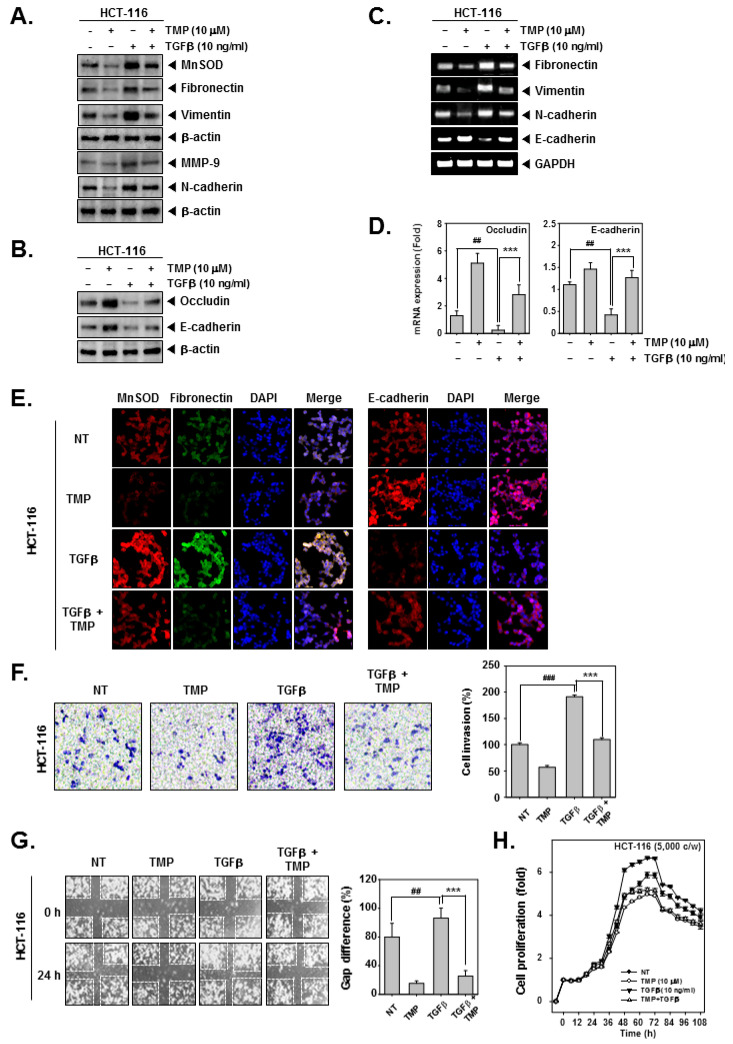
Effect of TMP on TGFβ-stimulated invasion and migration in HCT-116 cells. HCT-116 cells were treated with 10 μM of TMP. (**A**,**B**) the effect of TMP on TGFβ-induced EMT was examined by Western blot analysis. (**C**) Total RNA was isolated from cells, and the expression of indicated transcripts was analyzed by RT-PCR. (**D**) mRNA levels of occludin and E-cadherin were evaluated by real-time PCR assay. (**E**) The effect of TMP on TGFβ (10 ng/mL)-induced expression of indicated proteins was analyzed using immunocytochemistry. DAPI was used to stain the nucleus. (**F**) The effect of TMP on TGFβ (10 ng/mL)-induced cell invasion was studied by Boyden chamber assay. (**G**) The effect of TMP on TGFβ (10 ng/mL)-induced cell migration was studied by wound healing assay. (**H**) The cell proliferation was examined by RTCA. All the experiments were repeated at least thrice. ### *p* < 0.001 vs. non-treated (NT) cells, ## *p* < 0.01 vs. non-treated (NT) cells, and *** *p* < 0.001 vs. TGF-β treated cells.

HCT-116 cells were treated with TGFβ and/or TMP followed by an examination of the expression of EMT-related proteins. TGFβ alone significantly induced the expression of fibronectin, vimentin, and N-cadherin and decreased the expression of occludin and E-cadherin (Figure 2A,B), whereas the combinational treatment of TGFβ and TMP significantly reduced the TGFβ-induced expression of fibronectin, vimentin, and N-cadherin and elevated the expression of occludin and E-cadherin (Figure 2A,B). Next, mRNA expression of epithelial and mesenchymal markers was examined in TGFβ and/or TMP-treated HCT-116 cells. Upon TGFβ stimulation, there was a significant increase in the transcripts of fibronectin, vimentin, and N-cadherin with an evident decline in the levels of E-cadherin transcript (Figure 2C). Thereafter, the expression of occludin and E-cadherin under the influence of TMP and TGFβ was examined by real-time PCR. It was observed that mRNA expression of occludin and E-cadherin in TGFβ-stimulated cells was decreased, whereas TMP significantly increased the mRNA levels of both of them (Figure 2D). The altered expression of transcripts observed upon TGFβ stimulation was reversed when cells were treated with TMP. GAPDH was used as input control. Additionally, the expression of MnSOD, fibronectin, and E-cadherin was examined in HCT-116 cells treated with TGFβ and/or TMP using immunofluorescence analysis. The expression of MnSOD and fibronectin was induced upon TGFβ treatment with a parallel decrease in the expression of E-cadherin (Figure 2E). The combination of TGFβ and TMP reversed the effects that were observed in cells treated with TGFβ alone.

### 3.6. TMP Reduces the Migration, Invasion, and Proliferation of TGFβ-Treated Colon Cancer Cells

The effect of treatment of TGFβ and/or TMP on migration and invasion of HCT-116 cells was examined. TGFβ treatment effectively induced the migration and invasion of HCT-116 cells, whereas the treatment of TGFβ and TMP significantly abrogated the TGFβ-driven cell motility (Figure 2F,G). Additionally, the cell proliferation in TGFβ-treated colon cancer cells was measured using the RTCA DP instrument (Roche Diagnostics GmbH, Germany). TMP imparted an antiproliferative effect in untreated and TGFβ-treated HCT-116 cells in a time-dependent fashion (Figure 2H). These results indicate that TMP may hinder the movement of cancer cells by abrogating TGFβ signaling cascade.

### 3.7. TMP Suppressed the Activation of Constitutive/TGFβ-Stimulated PI3K/Akt/mTOR and MAPK Signaling Pathways in Colon Cancer Cells

**Figure 3:**
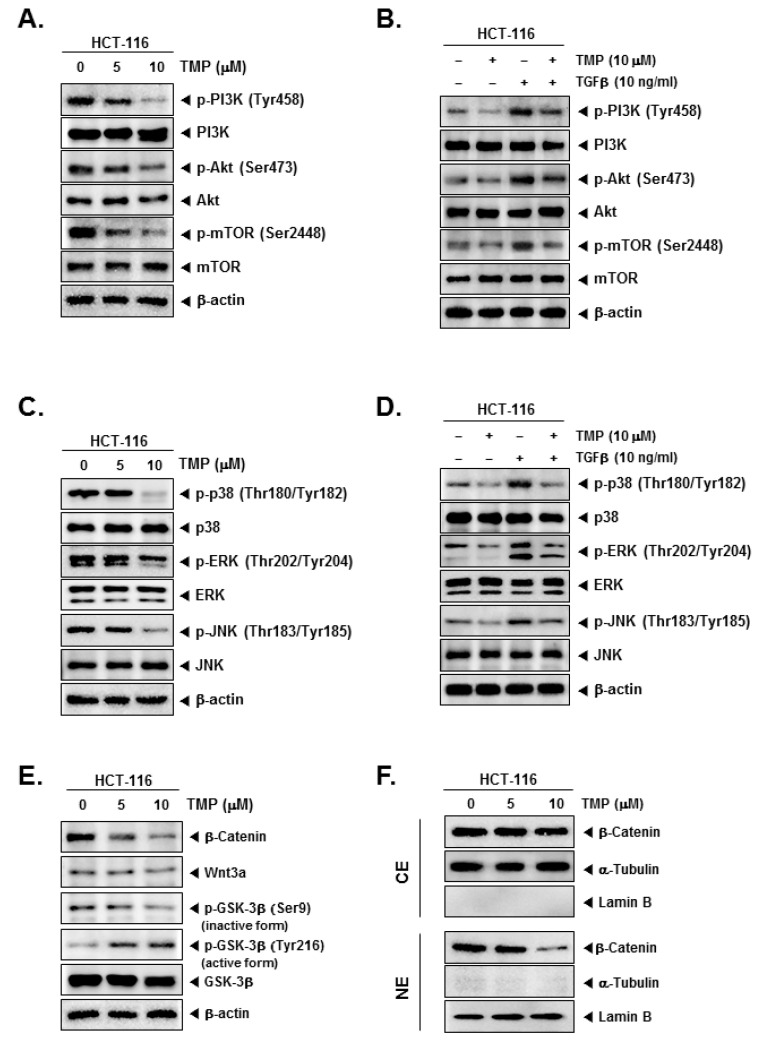
Effect of TMP on TGFβ-induced PI3K/Akt/mTOR, MAPK, and Wnt/β-catenin pathways activation in HCT-116 cells. (**A**,**C**,**E**) HCT-116 cells were seeded and treated with TMP (0, 5, 10 μM) for 6 h. The cell lysate was prepared with an equal amount of proteins. The expression of indicated proteins was examined by Western blot analysis. (**B**,**D**) HCT-116 cells were seeded and treated with TMP (0, 5, 10 μM) for 3 h, and TGFβ (10 ng/mL) for 3 h. The cell lysate was prepared and subjected to Western blot analysis to analyze the expression of indicated proteins. (**F**) Cytoplasm extracts (CE) and nucleus extracts (NE) were extracted from TMP (0, 5, 10 μM, 6 h)-treated HCT-cells. β-catenin, α-Tublin, and Lamin B was probed and examined by Western blot analysis. Each experiment was repeated at least thrice independently.

Further, the effect of TMP on PI3K/Akt/mTOR and MAPK signaling pathways in uninduced and TGFβ-induced cells were examined. TMP dose-dependently reduced the uninduced and TGFβ-induced expression of phosphorylated PI3K, Akt, and mTOR in HCT-116 cells (Figure 3A,B). In another set of experiments, TMP was also found to reduce the uninduced and TGFβ-induced phosphorylation of MAPK (p38, ERK, and JNK) proteins in a dose-dependent fashion (Figure 3C,D).

### 3.8. TMP Modulates Wnt/GSK3/β-Catenin Pathway in Colon Cancer Cells

The effect of TMP on Wnt/GSK3/β-catenin pathway proteins in HCT-116 cells was examined. TMP reduced the levels of β-catenin, Wnt3a, and phosphorylation of GSK-3β (Ser9, inactive form) in a dose-dependent manner (Figure 3E), whereas phosphorylation of GSK-3β (Tyr216, active form) was increased dose-dependently, indicating the activation of the pathway (Figure 3E). In parallel, levels of β-catenin were examined in nuclear and cytoplasmic extracts of TMP-treated HCT-116 cells. The reduction in the β-catenin levels was observed in the nuclear extract of TMP-treated cells (Figure 3F). α-Tubulin and lamin B were used as input controls of cytoplasmic and nuclear extracts, respectively.

### 3.9. MnSOD Can Modulate the Suppressive Effects of TMP on EMT in Colon Cancer Cells

**Figure 4:**
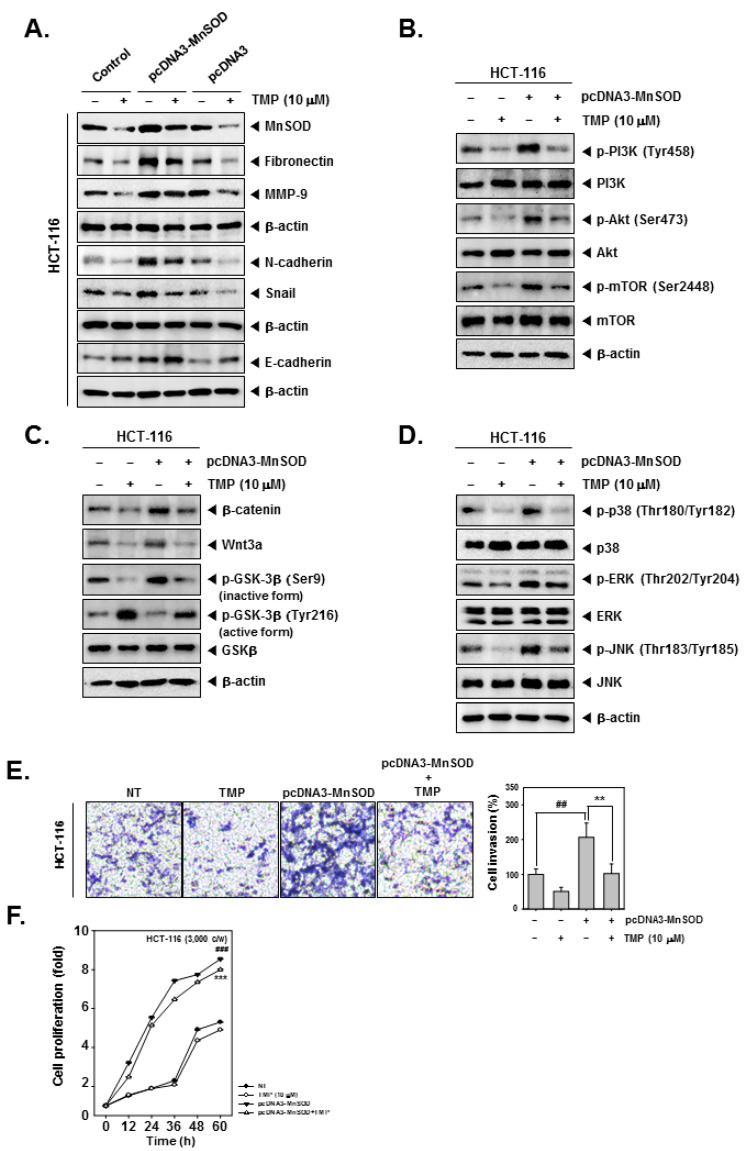
Effects of TMP on MnSOD overexpressed invasion, and migration in HCT-116 cells. HCT-116 cells (5 × 10^4^ cells/well) were transfected with pcDNA3-MnSOD for 24 h. (**A**) Then, cells were treated with TMP (10 μM) for 24 h. Indicated EMT markers were evaluated by Western blot analysis. (**B**) The cells were treated with TMP (10 μM) for 6 h, then PI3K/Akt/mTOR activation was analyzed by Western blot analysis. (**C**,**D**) TMP (10 μM, 6 h) treated cells were evaluated Wnt/β-catenin and MAPK signals by Western blot analysis. (**E**) Invasion activity of MnSOD overexpressed cells was evaluated by Boyden chamber assay. The cells were incubated for 5 h and stained in blue for observation. (**F**) MnSOD-overexpressed HCT-116 cells were treated with TMP (10 μM), then cell proliferation was evaluated by RTCA. All the experiments were repeated at least thrice independently. ### *p* < 0.001 vs. non-treated (NT) cells, ## *p* < 0.01 vs. non-treated (NT) cells, and *** *p* < 0.001 vs. pcDNA3-MnSOD transfected cells. ** *p* < 0.01 vs. pcDNA3-MnSOD transfected cells.

The effect of TMP on the induced expression of MnSOD and EMT-related signaling networks was investigated in HCT-116 cells. For this, HCT-116 cells were transfected with pcDNA3-MnSOD to initiate an overexpression of constitutively active MnSOD. Overexpression of MnSOD resulted in the increased expression of fibronectin and N-cadherin and decreased expression of E-cadherin and Snail. TMP treatment resulted in marginal reversal of expression of EMT-related proteins in pcDNA3-MnSOD-transfected cells (Figure 4A). Furthermore, HCT-116 cells were transfected with pcDNA3-MnSOD and analyzed for the activation status of PI3K/Akt/mTOR, Wnt/GSK3/β-catenin, and MAPK pathway proteins. TMP treatment caused a reduction in activation of PI3K/Akt/mTOR, Wnt/GSK3/β-catenin, and MAPK pathway proteins in pcDNA3-MnSOD transfected cells (Figure 4B–D).

### 3.10. TMP Impedes MnSOD Induced Migration and Proliferation of Colon Cancer Cells

The effect of TMP on migration and proliferation of pcDNA3-MnSOD-transfected cells was examined. pcDNA3-MnSOD-transfected cells showed a high degree of cell migration and proliferation, and TMP treatment significantly reverted the migration and proliferation of colon cancer cells (Figure 4E,F), indicating the MnSOD-dependent functions are significantly counteracted by TMP.

### 3.11. TMP Inhibits Cell Invasion in MnSOD-Depleted Colon Cancer Cells

**Figure 5:**
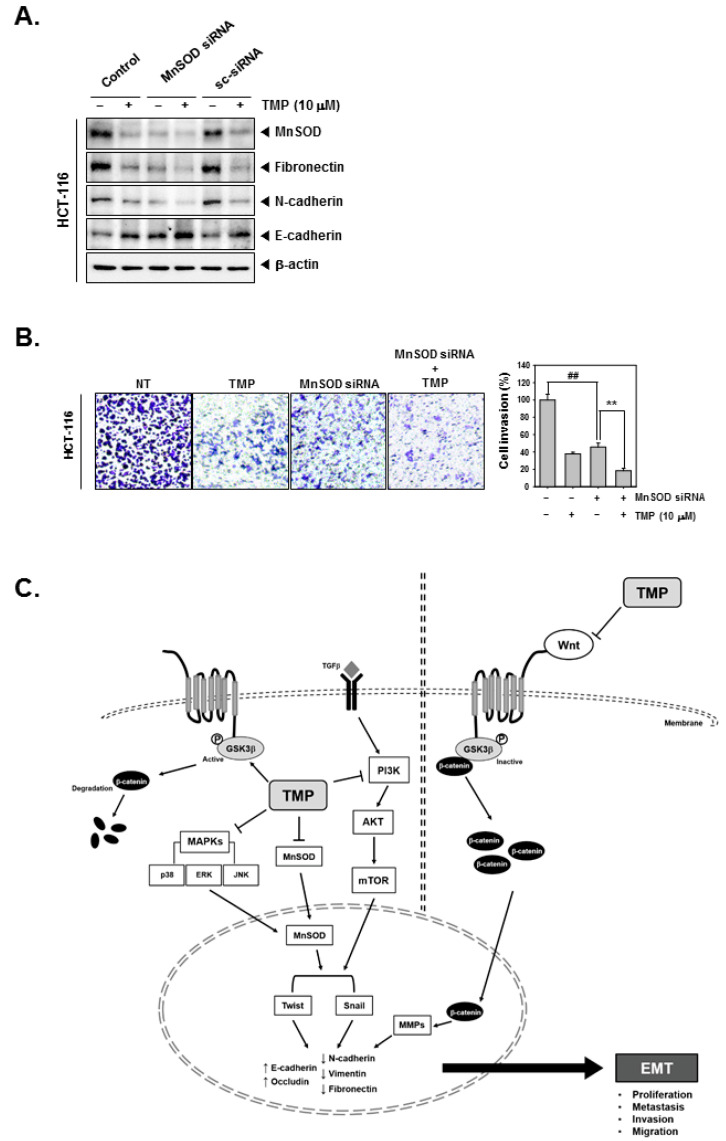
Effects of TMP and MnSOD knockdown on invasion and migration in HCT-116 cells. HCT-116 cells (5 × 10^4^ cells/well) were transfected with MnSOD siRNA for 24 h. (**A**) Then, the cells were treated with TMP (10 μM) for 24 h. Indicated EMT markers were evaluated by Western blot analysis. (**B**) Invasion activity of MnSOD knockdown cells was evaluated by Boyden chamber assay. The cells were incubated for 5 h and stained in blue for observation. (**C**) Schematic diagram of TMP in HCT-116 cells. All the experiments were repeated at least thrice independently. ## *p* < 0.01 vs. non-treated (NT) cells, and ** *p* < 0.01 vs. MnSOD siRNA transfected cells.

MnSOD was depleted using siRNA in HCT-116 cells, and then the cells were treated with TMP to analyze the cell invasion. As shown in the results, MnSOD knockdown followed by TMP treatment can significantly reduce the expression of fibronectin and N-cadherin while E-cadherin was induced (Figure 5A). Moreover, inhibition of cell invasion by MnSOD knockdown and TMP treatment in HCT-116 cells was observed (Figure 5B).

## 4. Discussion

TMP is a plant-derived secondary metabolite with anticancer activity against various types of cancer cells. However, its effect on EMT remains elusive. The objective of this study focused on determining the effect of TMP on the expression of MnSOD, EMT-related proteins, and the activation status of EMT-connected signaling events in colon cancer cells. The results of the present study demonstrated that TMP suppressed the constitutive and TGFβ-triggered EMT process and reduced the expression of MnSOD in a panel of colon cancer cells. Additionally, TMP-induced EMT inhibition was correlated with the abrogation of PI3K/Akt/mTOR, Wnt/GSK3/β-catenin, and MAPK signaling pathways (Figure 5C).

EMT is a critical event that triggers the activation of a broad range of signaling events, which are responsible for cancer progression [36,37]. We have previously demonstrated that the expression of MnSOD is positively correlated with breast cancer EMT score [15]. It was also demonstrated earlier that drug-resistant cancer cells display mesenchymal features with a significant elevation of MnSOD, and deletion of MnSOD leads to “turning off” of the EMT process [15]. The colon cancer cells that are under the present investigation showed good expression of MnSOD, which was significantly decreased upon TMP treatment. Therefore, we hypothesized that TMP may interfere with the EMT program in colon cancer cells. During the EMT process, cancer cells withdraw the epithelial markers (such as occludin and E-cadherin) and express mesenchymal markers (fibronectin, vimentin, and N-cadherin), which make the cancer cells mobile and disease aggressive [38]. Elevated expression of N-cadherin is correlated with metastasis and dismal prognosis in colorectal cancers [39]. Loss of occludin and E-cadherin is associated with cell motility and a poor clinical outcome in human cancers [40,41]. We investigated the expression of epithelial and mesenchymal markers in untreated and TMP-treated colon cancer cells through western blotting and immunofluorescence assays. We observed the elevated expression of fibronectin, vimentin, MMP-9, and N-cadherin with a parallel reduction in the levels of occludin and E-cadherin in the untreated cells indicating that the tested cancer cells present mesenchymal-like characteristics. TMP treatment reduced the expression of mesenchymal markers and re-established the expression of epithelial markers suggesting that TMP may abrogate the EMT program in the cytoplasm. As per the western blotting results, TMP induced the expression of E-cadherin, but we also observed that adhering junction is not functional since the immunoreactivity was not detected at cell boundaries as per the results of immunofluorescence assays. Additionally, transcripts of epithelial markers were upregulated, and mesenchymal markers were downregulated upon TMP treatment, indicating that the EMT process is being regulated at the level of transcription. MMPs contribute greatly to the metastatic ability of the cancer cells. Therefore, lowering of mesenchymal markers and MMPs must reduce the migration potential of cancer cells. Functionally, TMP imparted antimigration potential in colon cancer cells.

TGFβ has been endowed with tumor promoter as well as tumor suppressor functions depending on the tumor grade. The ability of TGFβ to induce prometastatic activity in various cancers has been well-documented. The activation of TGFβ in the tumor microenvironment generally promotes cancer cell survival [42]. Zhang and colleagues demonstrated that counteracting TGFβ signaling with an inhibitor in colon cancer results in the decrease of liver metastases and extends survival in an experimental metastasis model [43]. We subsequently investigated the effect of TMP on TGFβ-stimulated HCT116 cells. It was observed that TGFβ stimulation increased the levels of MnSOD, fibronectin, vimentin, MMP-9, and N-cadherin and decreased the levels of occludin and E-cadherin in colon cancer cells above the basal level, indicating the mesenchymal-phenotype of these cells is promoted by TGFβ. In line with our previous observation, TMP impeded the effects induced by TGFβ. Analysis of mRNA expression and immunofluorescence supported the data obtained in western blotting. The transcript levels were modulated in the same trend as per the related protein expression. Functionally, characterization also revealed that TMP imparted antimigration potential in colon cancer cells induced with TGFβ.

TGFβ and c-Met signaling promote the operation of oncogenic cascades including PI3K/Akt/mTOR, Wnt/GSK3/β-catenin, MAPK, and other downstream pathways [44,45]. Therefore, inhibition of TGFβ could act as a common upstream point to target a broad range of signaling events that are associated with tumor initiation and progression. Our results demonstrated that TMP downregulates the uninduced/TGFβ-induced phosphorylation of key proteins of PI3K/Akt/mTOR (such as PI3K, Akt, and mTOR), MAPKs (such as p38, ERK, and JNK), and Wnt/GSK3/β-catenin (such as GSK3β) signaling pathways. The reduction in the nuclear levels of β-catenin upon TMP treatment was also noted. The nuclear translocation of β-catenin enhances the transcriptional activity and subsequent events associated with tumorigenesis [46,47]. Yu and colleagues made a similar observation in the downregulation of all these pathways upon treatment with dictamnine (c-Met inhibitor) in lung cancer cells [48]. Further, we were interested to learn about the effect of forced expression of MnSOD by transfecting colon cancer cells with pcDNA3-MnSOD. The transfection of cancer cells with pcDNA3-MnSOD significantly enhanced the expression of MnSOD, fibronectin, vimentin, MMP-9, and N-cadherin, and decreased the levels of E-cadherin in colon cancer cells above the basal level, indicating the mesenchymal-phenotype of these cells is promoted by when MnSOD levels go high in the cell. Snail, Twist, and Zeb are the key transcription factors associated with the EMT process, and there was a clear elevation in the levels of Snail in pcDNA3-MnSOD-transfected cells. The expression of all these proteins was reversed when the transfected cells were treated with TMP, indicating that constitutive activation of MnSOD-related oncogenic proteins may be counteracted by TMP. Interestingly, opposite results were obtained upon knockdown of MnSOD by using si-RNA in colorectal cancer cells.

Overall, we have previously demonstrated that PI3K/Akt signaling pathway is critically involved in the EMT process and counteracting this signaling results in the reversal of EMT. PTEN is the negative modulator of the PI3K/Akt signaling pathway, and inhibition of PTEN activity results in deregulated activation of the PI3K/Akt cascade. Previous reports suggest that MnSOD elevates the level of H_2_O_2_, which in turn inhibits PTEN activity thereby contributing to the deregulated activation of PI3K/Akt signaling in cancer cells. Qiu and colleagues have demonstrated that the blockade of MnSOD/FoxM1 signaling in head and neck squamous cell carcinoma cells results in the inhibition of expression of EMT-related transcription factors [17]. This could be due to the modulation of the MnSOD/PTEN/PI3K/Akt/FOXO/FoxM axis. Additionally, we used the keyword “tetramethylpyrazine” and searched in Pubmed and found 1254 results (accessed on 4 March 2022). Later, we used the keyword “tetramethylpyrazine cancer” and found 107 results. The results presented that TMP imparts anticancer activity through the diverse mode of actions and targets (possibly lacks specificity). It may therefore be a typical “frequent binder”, often encountered in natural compounds, but not very helpful when it comes to elucidating the mechanisms of action. The data relating to EMT could be due to these pleiotropic effects of TMP. This also emphasizes the fact that it is difficult to identify the precise target of TMP as seen many in natural compounds. In conclusion, we have demonstrated that TMP can interfere with the TGFβ-induced EMT process through the downregulation of expression of MnSOD, EMT-related proteins, and oncogenic pathways such as PI3K/Akt/mTOR, Wnt/GSK3/β-catenin, and MAPK pathways. We have attempted to establish the link between TGFβ signaling, MnSOD expression, and the signaling cascades for the first time in literature. We conclude that the inhibition of the TGFβ-induced EMT process could serve as a potential therapeutic strategy to counteract the cancer progression.

## Data Availability

The data presented in this study are available on request from the corresponding author.
